# Idiopathic Pseudoaneurysm of the Radial Artery: A Case Successfully Diagnosed Using Point-of-Care Ultrasound and Treated With Endovascular Therapy

**DOI:** 10.7759/cureus.85654

**Published:** 2025-06-09

**Authors:** Masataka Miyamoto, Daisuke Mizu, Takuya Maekura

**Affiliations:** 1 Department of Emergency Medicine, Japanese Red Cross Osaka Hospital, Osaka, JPN; 2 Department of Radiology, Japanese Red Cross Osaka Hospital, Osaka, JPN

**Keywords:** coil embolization, endovascular treatment, idiopathic pseudoaneurysm, point-of-care ultrasound, soft tissue disorder

## Abstract

Pseudoaneurysms of the radial artery often occur in association with catheterization procedures or trauma, and idiopathic cases are rare. Point-of-care ultrasonography (POCUS) is effective for diagnosis; however, the effectiveness of endovascular treatment for pseudoaneurysms remains unclear. A 64-year-old male patient presented to the emergency department with pain and swelling of the left forearm and hand. Symptoms appeared seven days prior and were treated with antibiotics, but there was no improvement. POCUS revealed a yin-yang sign, and contrast-enhanced computed tomography revealed a pseudoaneurysm of the radial artery. Endovascular treatment was performed. Idiopathic radial artery pseudoaneurysms are rare, and differentiating them from other soft tissue diseases is important. Emergency physicians should be aware that POCUS is effective for the rapid diagnosis of idiopathic radial artery pseudoaneurysms and that endovascular treatment is less invasive and may be effective.

## Introduction

Radial artery pseudoaneurysms are a rare condition that is most commonly associated with catheter procedures or trauma [1-3]. Idiopathic cases are extremely rare and lack a history of contributing factors, necessitating differentiation from other soft tissue disorders. Misdiagnosis can lead to unnecessary puncture or incision of the pseudoaneurysm [4,5], and delayed diagnosis and treatment can result in complications such as delayed hemorrhage, thrombosis, or neurological deficits [6,7]. Physical examination is unreliable for diagnosing pseudoaneurysms, while point-of-care ultrasonography (POCUS) is considered effective [6,8]. Surgical treatment, ultrasound-guided compression, and thrombin injection have been used as treatments [1,4-7,9]; however, the efficacy of endovascular treatment remains unclear. We report a case of idiopathic pseudoaneurysm of the radial artery initially treated as a soft tissue infection that was diagnosed using POCUS and managed with endovascular treatment. This case highlights the diagnostic challenges of idiopathic radial artery pseudoaneurysms. Furthermore, it highlights the validity and importance of the aggressive use of POCUS.

## Case presentation

A 64-year-old male patient with a history of cerebral infarction and paroxysmal supraventricular tachycardia on edoxaban presented with pain and swelling of the left forearm and hand without any apparent cause. There were no sequelae of cerebral infarction and no history of atrial fibrillation. The patient visited a local clinic seven days earlier and was treated with cefcapene pivoxil hydrochloride hydrate for a presumed soft tissue infection. As the symptoms did not improve, the patient presented to the emergency department.

On examination, the patient had a Glasgow Coma Scale score of 15, blood pressure of 123/70 mmHg, heart rate of 79 beats/minute, oxygen saturation level of 98% on room air, respiratory rate of 16 breaths/minute, and temperature of 36.2°C. A detailed medical history revealed no trauma to the forearm. Physical examination revealed swelling, pain, and warmth in the distal left forearm without erythema or pulsatility. There were no abnormalities in hand movements or sensations. Blood tests showed a mild elevation in white blood cell count and C-reactive protein levels, with normal coagulation function (Table 1).

**Table 1:** Patient laboratory data at the emergency department TP: total protein, Alb: albumin, AST: aspartic aminotransferase, ALT: alanine aminotransferase, BUN: blood urea nitrogen, Cr: creatinine, CK: creatine kinase, Glu: glucose, CRP: C-reactive protein, CBC: complete blood count, WBC: white blood cell, Hb: hemoglobin, Plt: platelet, APTT: activated partial thromboplastin time

Biochemistry	Value	Normal range
TP	7.5 g/dL	6.5-5.8 g/dL
Alb	4.1 g/dL	3.9-4.2 g/dL
AST	16 IU/L	8-40 IU/L
ALT	14 IU/L	8-40 IU/L
BUN	18.8 mg/dL	8-20 mg/dL
Cr	0.88 mg/dL	0.4-0.8 mg/dL
CK	48 IU/L	50-170 IU/L
Glu	125 mg/dL	70-110 mg/dL
CRP	2.15 mg/dL	0-0.5 mg/dL
CBC/coagulopathy	Value	Normal range
WBC	136×10²/μL	39-98×10²/μL
Hb	14.5 g/dL	11.1-15.1 g/dL
Plt	22.7×10⁴/μL	13-37×10⁴/μL
APTT	32 seconds	60-110 seconds

**Figure 1:**
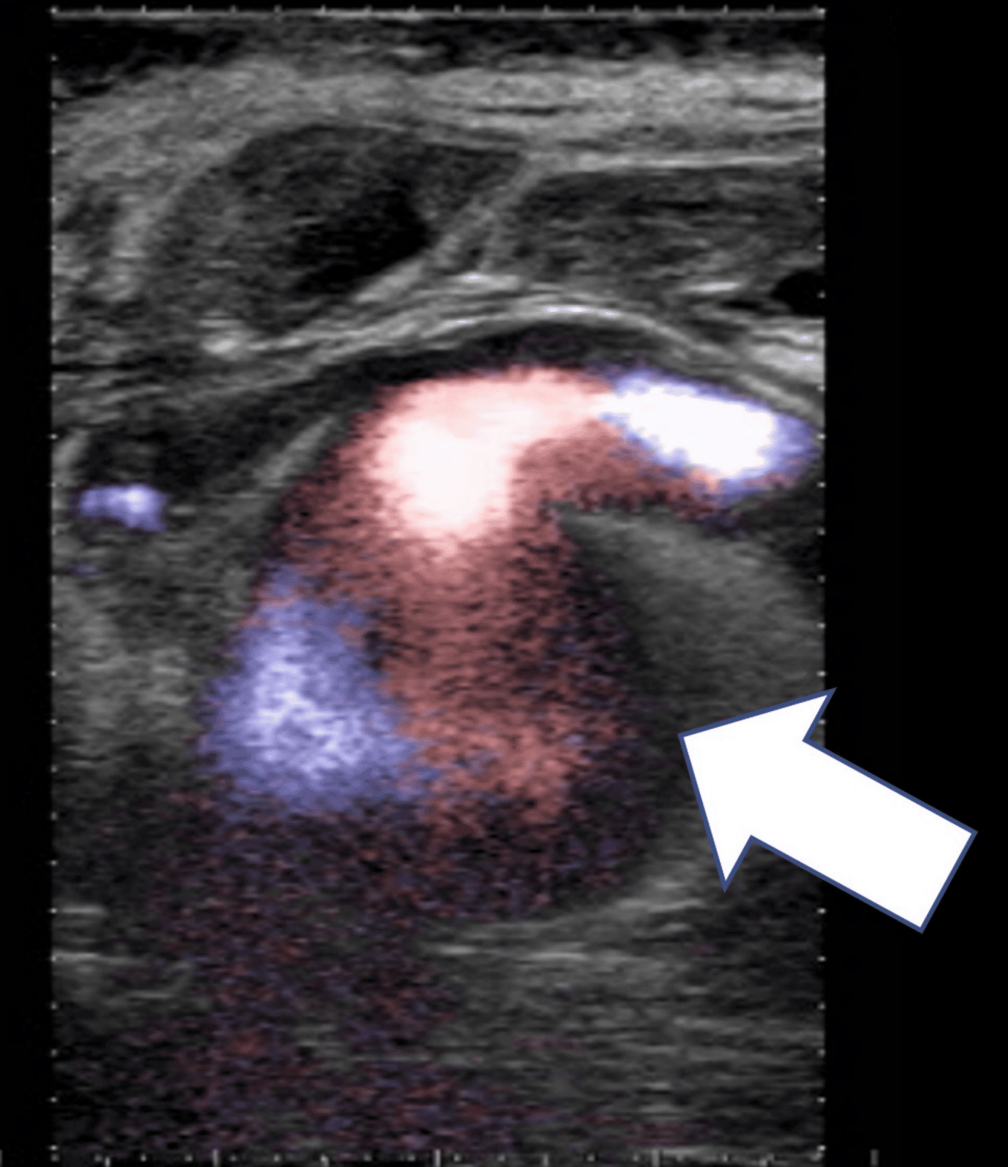
Point-of-care ultrasound showing the yin-yang sign (white arrow).

POCUS revealed findings suggestive of an aneurysm at the swelling site. Doppler flow showed bidirectional flow within the aneurysm (yin-yang sign), indicating a pseudoaneurysm (Figure 1).

**Figure 2:**
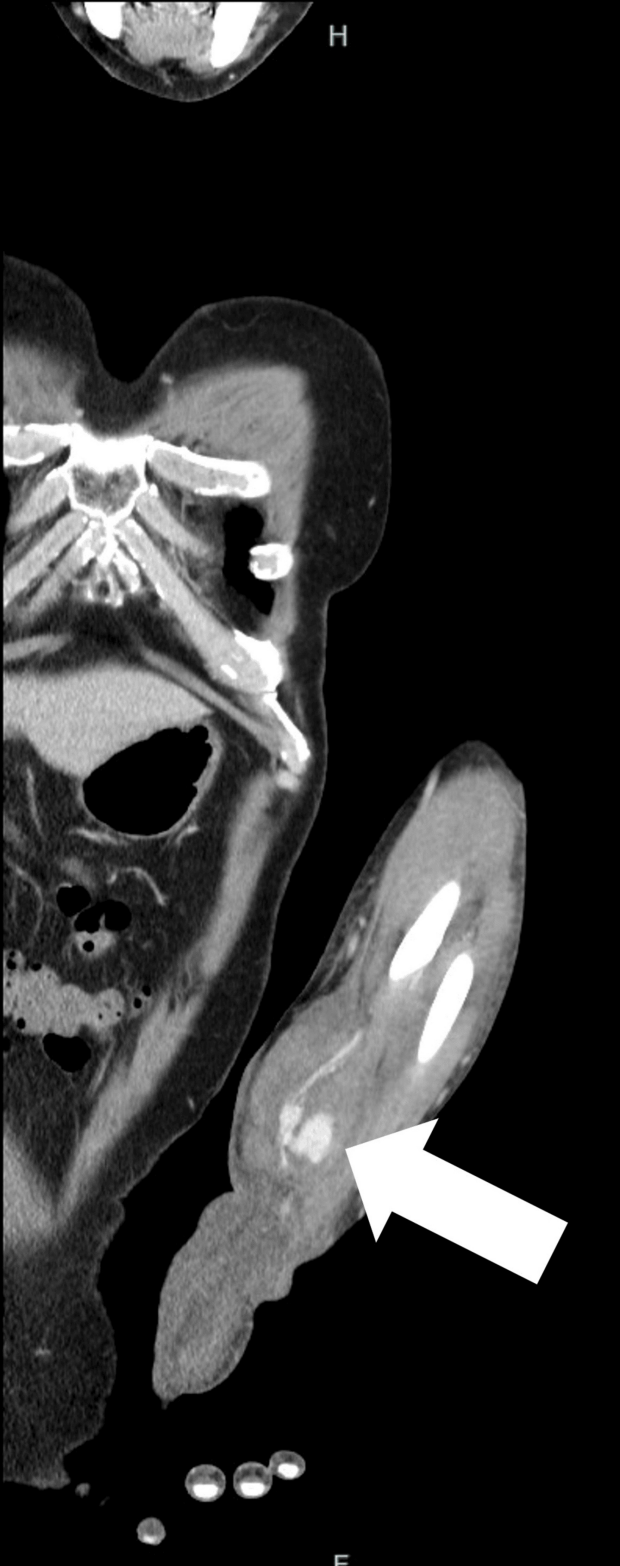
Contrast-enhanced computed tomography of the left forearm: a 29-mm pseudoaneurysm was seen that was continuous with the radial artery (white arrow).

Contrast-enhanced computed tomography confirmed the diagnosis of a radial artery pseudoaneurysm (Figure 2).

**Figure 3:**
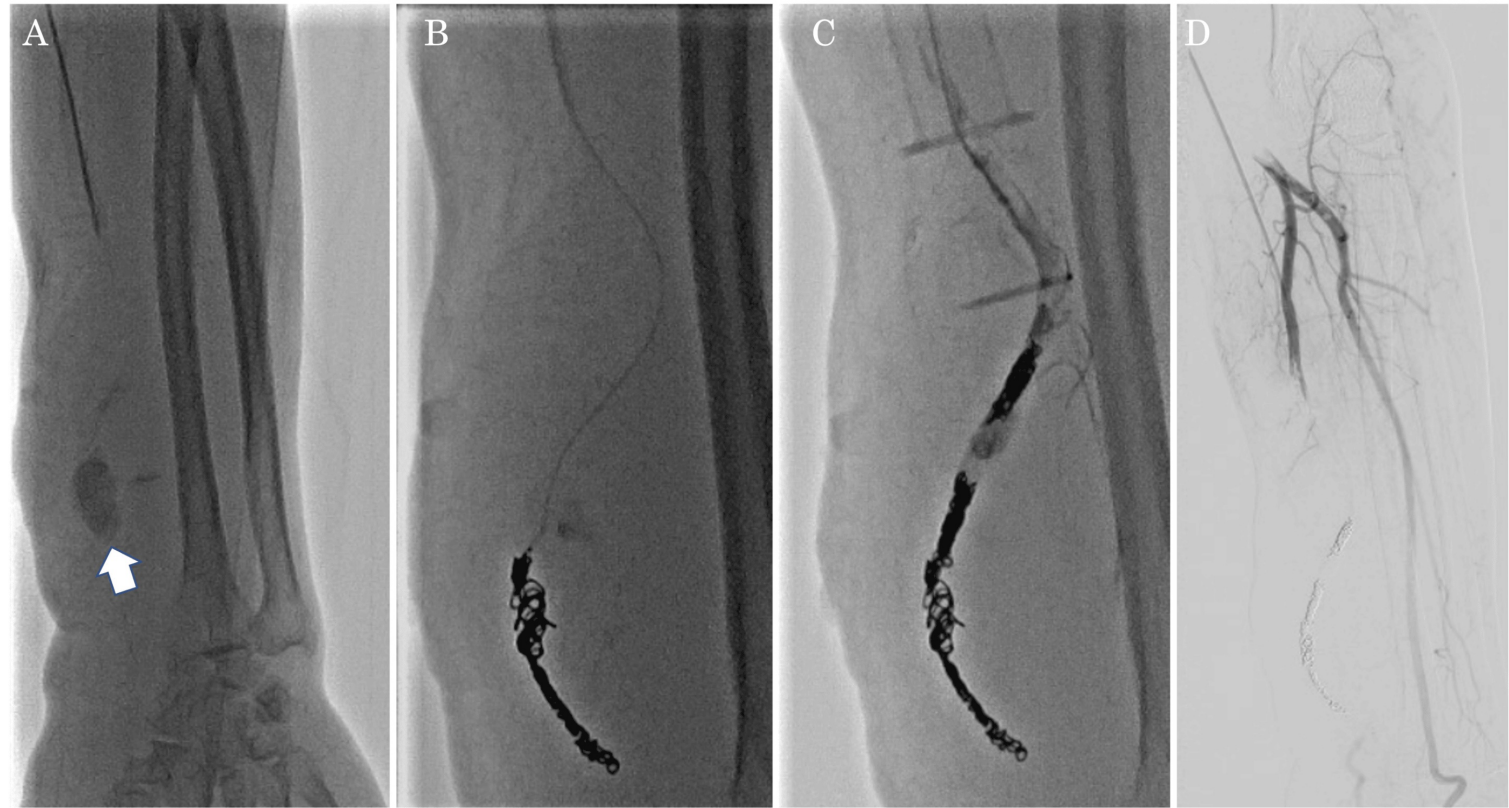
A: Pseudoaneurysm rupture was confirmed before coil embolization (white arrow). B: Coil embolization was performed. C and D: After treatment, the disappearance of blood flow was confirmed.

Edoxaban was suspended, but an antagonist was not administered since there was no bleeding. The patient was admitted and underwent ultrasound-guided direct compression of the pseudoaneurysm for one hour and 30 minutes, followed by continuous compression for 24 hours using a fixation device. As the pain and swelling did not improve, endovascular treatment was performed on the second day after admission. The proximal left radial artery was punctured. A microballoon catheter was inflated proximal to the pseudoaneurysm, confirming the cessation of blood flow within it. Embolized with two Target XL 360 Soft 2 mm × 6 cm, three 3 mm × 9 cm, seven C-Stopper 14 2 mm × 6 cm, one Target XL 4 mm × 12 cm, and two C-Stopper 14 2 mm × 6 cm electrical breakaway coils. Blood flow from the ulnar artery was preserved, and the balloon was left in place to complete the procedure. However, subsequent ultrasonography showed recurrent blood flow within the pseudoaneurysm, and coil embolization was performed (Figure 3).

Blood flow within the pseudoaneurysm was successfully eliminated, and the patient was discharged on the eighth day of admission. Edoxaban was resumed after discharge from the hospital. We confirmed at an outpatient visit two weeks after the patient's discharge that there was no recurrence of symptoms. Subsequent follow-up was conducted by the patient's primary care physician.

## Discussion

This case describes the misdiagnosis of an idiopathic radial artery pseudoaneurysm as a soft tissue infection that was promptly diagnosed using POCUS and treated endovascularly. Radial artery pseudoaneurysms should be suspected in cases of forearm swelling and pain following trauma or medical puncture procedures [10], although idiopathic pseudoaneurysms are rare. Typically, pseudoaneurysms present as swelling, pain, and a pulsatile mass [4-7]. However, in this case, pulsatility was not palpable, possibly because of insufficient physical examination results.

Physical examination is unreliable for the diagnosis of pseudoaneurysms, and there have been reports of misdiagnosis as infections in the upper limbs [11]. When soft tissue swelling and pain are present, pseudoaneurysms should be considered in the differential diagnosis. POCUS is effective in the diagnosis of pseudoaneurysms, which are characterized by expansile pulsatility, bidirectional blood flow within the vessel (known as the yin-yang sign), and hematoma with variable echogenicity [6,12]. Although POCUS is not the gold standard for diagnosis, it is a non-invasive and immediate tool with high sensitivity and specificity [12]. The aggressive use of POCUS is important for the early diagnosis of pseudoaneurysms. The onset was characterized by acute pain and swelling without redness or fever. Therefore, there were no preceding symptoms suggestive of infection, and the possibility of infectious aneurysm from cellulitis was considered low.

The efficacies of ultrasound-guided compression, thrombin injection, and surgical treatment have been reported [6,9,12]. However, the effectiveness of endovascular treatment remains unclear. The success rate of direct compression is reportedly lower when anticoagulants are used [13], which was also observed in this patient. In this case, coil embolization ensured hemostasis, and no complications were observed. As shown in previous studies, this is considered an effective and safe treatment option [14]. Further studies are needed to validate the utility of endovascular treatment for radial artery pseudoaneurysms.

## Conclusions

Idiopathic pseudoaneurysms of the radial artery are rare but should be considered in the differential diagnosis of soft tissue disorders. Physical examination alone is inadequate, and the active use of POCUS can lead to early diagnosis. Although many treatment options are available, emergency physicians must be aware of their efficacy and uncertainty. Endovascular treatment can be safe and highly effective, although further research is needed.
